# Targeting of interleukin-13 receptor α2 for treatment of head and neck squamous cell carcinoma induced by conditional deletion of TGF-β and PTEN signaling

**DOI:** 10.1186/1479-5876-11-45

**Published:** 2013-02-19

**Authors:** Bradford Hall, Hideyuki Nakashima, Zhi-Jun Sun, Yuki Sato, Yansong Bian, Syed R Husain, Raj K Puri, Ashok B Kulkarni

**Affiliations:** 1 Functional Genomics Section, Laboratory of Cell and Developmental Biology, National Institute of Dental and Craniofacial Research, National Institutes of Health, 30 Convent Drive, Building 30, Room 130, Bethesda, MD, USA; 2 Tumor Vaccines and Biotechnology Branch, Division of Cellular and Gene Therapies, Center for Biologics Evaluation and Research, U.S. Food and Drug Administration, Bethesda, MD, USA; 3 Tumor Biology Section, Head and Neck Surgery Branch, National Institute on Deafness and Other Communication Disorders, National Institutes of Health, Bethesda, MD, USA

**Keywords:** IL-13 receptor, Immunotoxin, TGF-β, PTEN, HNSCC

## Abstract

**Background:**

The sixth leading class of cancer worldwide is head and neck cancer, which typically arise within the squamous epithelium of the oral mucosa. Human head and neck squamous cell carcinoma (HNSCC) is known to be difficult to treat and has only a 50% five-year survival rate. With HNSCC, novel therapeutics are needed along with a means of rapidly screening anti-cancer agents *in vivo*, such as mouse models.

**Methods:**

In order to develop new animal models of cancer to test safety and efficacy of novel therapeutic agents for human HNSCC, tumors resembling clinical cases of human HNSCC were induced in the head and neck epithelium of a genetically engineered mouse model. This mouse model was generated by conditional deletion of two tumor suppressors, Transforming Growth Factor-β Receptor 1 (TGFβRI) and Phosphatase and Tensin homolog (PTEN), in the oral epithelium. We discovered that the tumors derived from these *Tgfbr1/Pten* double conditional knockout (2cKO) mice over-expressed IL-13Rα2, a high affinity receptor for IL-13 that can function as a tumor antigen. To demonstrate a proof-of-concept that targeted therapy against IL-13Rα2 expression would have any antitumor efficacy in this spontaneous tumor model, these mice were treated systemically with IL-13-PE, a recombinant immunotoxin consisting of IL-13 fused to the *Pseudomonas* exotoxin A.

**Results:**

*Tgfbr1/Pten* 2cKO mice when treated with IL-13-PE displayed significantly increased survival when compared to the untreated control mice. The untreated mice exhibited weight loss, particularly with the rapid onset of tongue tumors, but the treated mice gained weight while on IL-13-PE therapy and showed no clinical signs of toxicity due to the immunotoxin. Expression of IL-13Rα2 in tumors was significantly decreased with IL-13-PE treatment as compared to the controls and the number of myeloid-derived suppressor cells (MDSC) was also significantly reduced in the spleens of the IL-13-PE treated mice.

**Conclusions:**

Our study demonstrates that the *Tgfbr1/Pten* 2cKO mouse model of human HNSCC is a useful model for assessing antitumor activity of new cancer therapeutic agents, and that IL-13-PE has therapeutic potential to treat human head and neck cancer.

## Introduction

In order to test new therapies for the prevention and treatment of head and neck squamous cell carcinoma (HNSCC), we generated a novel mouse model that mimics clinical cases of human head and neck cancer [1]. Our mouse model of HNSCC allows for conditional deletion of two important tumor suppressors in the oral epithelium: Transforming Growth Factor-β Receptor 1 (TGFβRI), an inhibitor of epithelial proliferation, and phosphatase and tensin homolog (PTEN), an enzyme that negatively regulates PI3K/Akt signaling to prevent uncontrolled cell growth. Patients with human HNSCC often display alterations in the cellular signaling pathways associated with these two tumor suppressors [2-4]. Upon deletion of both TGFβRI and PTEN, the resulting double conditional knockout (2cKO) mice develop papillomas as early as 4 weeks after tumor induction, and these tumors progress to squamous cell carcinomas with 100% penetrance. The majority of the *Tgfbr1/Pten* 2cKO mice develop tumors on the epithelium of the tongue, although tumors also form elsewhere on the head and neck epithelium such as the ears and muzzle area. The tumors in the *Tgfbr1/Pten* 2cKO mice display many of the same biochemical alterations that are common to human HNSCC, particularly with regard to upregulation of inflammatory cytokines that promote tumor growth and proliferation.

Since HNSCC accounts for about 8% of newly diagnosed cancers worldwide [5] with an overall 5-year survival rate of only 40–50% in patients despite recent advances in multimodality therapy [6], novel therapeutic agents are needed that will increase overall survival while avoiding the toxicities associated with current radiotherapy and chemotherapy. We have previously reported that IL-13Rα2, a receptor that binds IL-13 with high affinity, is uniquely overexpressed on some human head and neck cancers. The expression of the IL-13 receptors has been shown to be significantly higher in HNSCC than in normal tissues with a tissue array. In particular, about 33% of human HNSCCs display moderate to high-level expression of IL-13Rα2 [7]. The expression of IL-13Rα2, a unique receptor that binds IL-13 with high affinity and involved in signaling in cancer cells, is over expressed in cancer while its expression is mostly absent or low on other tissues except expression in testicular tissue [8]. We therefore, examined tumors from the *Tgfbr1/Pten* 2cKO mice for expression of IL-13Rα2 in order to establish any further link between our mouse model and human cases of HNSCC. We discovered that this receptor is overexpressed within the squamous cell carcinomas of the mice. With this knowledge of IL-13Rα2 upregulation in the tumors, we hypothesized that our mouse model could be used to study targeted therapy against this cancer marker.

One therapy that can be used to target expression of the IL-13Rα2 tumor antigen involves the use of a novel recombinant immunotoxin, designated IL-13-PE, which consists of the IL-13 cytokine fused to the *Pseudomonas* exotoxin A (PE) [9]. IL-13-PE has been shown to have cytotoxic effects against head and neck cancer cell lines that are either positive for IL-13Rα2 or are stably transfected to express IL-13Rα2. Anti-tumor activity was demonstrated with IL-13-PE in an immunodeficient xenograft mouse model using these human HNSCC cell lines and the immunotoxin treatment were well-tolerated by the mice [10]. With the sensitivity of these HNSCC cell lines to IL-13-PE, we decided to test this immunotoxin treatment in a genetically engineered mouse model of head and neck cancer, the *Tgfbr1/Pten* 2cKO mice. Because secretion of IL-13 can influence tumor suppressor cells including myeloid derived supressor cells leading to immunoevasion [11,12], testing IL-13-PE in the immunocompetent *Tgfbr1/Pten* 2cKO mice will allow us to study the effects of this treatment on the tumor microenvironment, particularly the interactions with the immune system. Also, there is a known interplay between IL-13 and TGF-β signaling [13,14], so it will additionally be interesting to examine IL-13-PE treatment in a context where, similar to a subset of human HNSCC patients, the cancer cells are unaffected by the growth inhibitory effects of TGF-β1 and can thereby utilize this cytokine to create a tumor-promoting microenvironment. So, HNSCC was induced in the *Tgfbr1/Pten* 2cKO mouse model and mice were dosed with IL-13-PE to determine the therapeutic effect of this treatment. The *Tgfbr1/Pten* 2cKO mice that received IL-13-PE treatment had significantly increased longevity as compared to the controls. These results suggest that our spontaneous immunocompetent head and neck tumor model is useful for testing cancer therapeutics and that IL-13-PE could be a useful treatment regimen for inhibiting the growth of human HNSCC.

## Materials and methods

### Tgfbr1/Pten 2cKO mice

The *Tgfbr1/Pten* 2cKO mice (*K14-CreER*^*tam*^*; Tgfbr1f/f/Ptenf/f*) were generated as previously described [1]. Essentially, tamoxifen (200 μl at a concentration of 10 μg/μl in corn oil) was applied to the oral cavity of 5- to 7-week-old *K14-CreER*^*tam*^*; Tgfbr1f/f/Ptenf/f* mice to induce the *K14-CreER*^*tam*^ transgene which causes deletion of TGFβRI and PTEN in the head and neck epithelium. Tumors developed in 100% of the mice as early as 4 weeks after conditional deletion of these tumor suppressors. When the lesions reached 2 cm, or became ulcerated, the animals were euthanized and a necropsy was performed. Mice were also euthanized in the survival studies if animals exhibited any signs of excessive weight loss (cachexia), typically due to tongue tumors that obstruct proper eating. All care to the mice was given in compliance with the National Institutes of Health guidelines on the use of laboratory and experimental animals, and the studies were approved by the National Institute of Dental and Craniofacial Research (NIDCR) Animal Care and Use Committee (ACUC).

### IL-13-PE treatment

A recombinant fusion IL-13 immunotoxin termed as IL-13-PE, consisting of human IL-13 and a mutated form of *Pseudomonas* exotoxin, was purified in our laboratory as described previously [9]. Since human IL-13 binds to murine cells, this fusion protein was used in murine experiments [15]. IL-13-PE was administered to the *Tgfbr1/Pten* 2cKO mice by i.p. injection in a volume of 500 μl (50 μg/kg) in 0.2% human serum albumin in PBS [10]. The i.p. schedule included two injections per day (b.i.d.) at a minimum interval of 6–8 hr on alternate days for two weeks (a total of 12 injections per mouse).

### Cell culture and protein synthesis inhibition assay

Primary cultures were established as previously described [16]. Excised tumors from the *Tgfbr1/Pten* 2cKO mice were enzymatically digested (10 mg/ml collagenase, 1 mg/ml hyaluronidase, and 0.5 mg/ml DNAse [Sigma-Aldrich, St. Louis, MO]) and the isolated tumor cells were cultured in RPMI1640 medium containing 10% FBS. After two to three passages, tumor cells were treated with IL-13-PE and cytotoxicity was measured. Essentially, 10^4^ cells were cultured with or without various concentrations of IL-13-PE for 22 hours in a leucine-free medium and then incubated with 1 μCi of ^3^H] leucine (NEN Research Products, Waltham, MA) for an additional 4 hours. Following these incubation times, the uptake of the radioactive leucine was measured using a Beta plate counter (Wallace, Waltham, MA).

### Histology and immunohistochemistry

Tumors along with control tissues such as buccal mucosa, tongue, spleen, lung, kidney, and liver were carefully dissected from the mice and fixed overnight in 10% buffered formalin. The tissues were embedded in paraffin and 5-μm sections were used for histopathological analysis. Tumor pathology was examined using slides stained with hematoxylin and eosin (H&E). For immunohistochemistry, sections were deparaffinized in xylene, rehydrated with descending grades of ethanol, blocked for 30 min, and incubated with primary antibody (see below) prepared in Background Reducing Antibody Diluent (Dako, Carpentaria, CA). Next, the sections were treated with the Rabbit on Rodent HRP-Polymer (Biocare, Concord, CA) or with a biotinylated secondary antibody (Vector Laboratories, Burlingame, CA) followed by treatment with the Vectastain ABC reagent (Vector Laboratories). Antibody complexes were detected using liquid DAB (Biogenex, Fremont, CA). Primary antibody dilutions are as follows: 1:100 dilution for anti-mouse IL-13Rα2 (R&D Systems, Inc., Minneapolis, MN), and 1:100 dilutions for phospho-Akt (Ser473) (D9E) antibody (Cell Signaling, Danvers, MA) and Anti-Mouse Ki-67 Antigen Clone TEC-3 (Dako, Carpentaria, CA).

### Western blot analysis

Tumors and normal epithelium from the buccal mucosa and tongue were collected from *Tgfbr1/Pten* 2cKO and *Tgfbr1f/f/Ptenf/f* mice, and protein lysates were generated using T-PER reagent (Pierce, Rockford, IL) with a complete mini protease inhibitor cocktail (Roche, Branchburg, NJ). About 50 μg of the extracted protein were denatured and then separated with electrophoresis on NuPAGE 4-12% Bis-Tris precast gels using XCell surelock Mini-Cell (Invitrogen, Carlsbad, CA). Proteins were then transferred onto a nitrocellulose membrane, blocked for 1 hour, and incubated with primary antibodies overnight. The attached antibodies were then visualized using a horseradish peroxidase-conjugated secondary antibody (Santa Cruz, Santa Cruz, CA) followed by chemiluminescence detection (Pierce, Rockford, IL). Subsequently, all blots were re-incubated with anti-Actin antibody (1:5000 dilution; Millipore, Billerica, MA) as a loading control. The following primary antibody dilutions were used: 1:250 for muIL-13Rα2 (R&D Systems, Inc., Minneapolis, MN) and 1:1000 for phospho-Akt (Ser473) (D9E), and, phospho-Stat3 (Tyr705) (D3A7), antibodies (Cell Signaling, Danvers, MA).

### Quantitative real-time PCR

Total RNA was extracted from the tongue tumors and adjacent control tongue tissue using 3 pairs of *Tgfbr1/Pten* 2cKO mice as well as a *Tgfbr1f/f/Ptenf/f* control. RNA was isolated using the miRNeasy Mini kit (Qiagen, Valencia, CA). For mRNA analysis, 0.5 μg of total RNA were reverse transcribed into cDNA and a tenth of this solution was then used per well with iQ SYBR Green Supermix (Bio-Rad, Hercules, CA) for real-time PCR (RT-PCR). Samples were run in quadruplicate using a Chromo4 PCR System (Bio-Rad). The IL-13 receptors (IL-13Rα2, IL-13Rα1, IL-4Rα) and a β-actin internal control were detected using QuantiTect Primers (Qiagen, Valencia, CA). RT-PCR to detect IL-13Rα2 expression on the primary tumor cultures was performed as previously described [16].

### Flow cytometric analysis

Splenocytes were isolated from three *Tgfbr1/Pten* 2cKO mice immediately following IL-13-PE treatment and then compared to the appropriate controls. Quantification of MDSCs (CD11b+ GR-1+) by FACS analysis was performed as previously described [17]. Essentially, 1 x 10^6^ splenocytes were evaluated using FITC-conjugated anti-CD11b and PerCP-Cy5.5–conjugated anti–Gr-1 Abs (e-Bioscience, San Diego, CA). All flow cytometric analyses were performed on a FACSCanto II (Becton Dickinson, San Jose, CA) flow cytometer.

### Statistical analysis

The data was analyzed for statistical significance using Graph Pad Prism version 5.00 for Windows (Graph-Pad Software Inc, La Jolla, CA). Survival curves were generated using the Kaplan-Meier method and compared using the Log-rank (Mantel-Cox) Test. Two-way ANOVA was used to compare the weight change between IL-13-PE treated and untreated groups. A two-tailed Student’s unpaired t test was used to determine statistical significance for all other comparisons including the determination of significant differences in receptor expression by real-time PCR, IL-13Rα2 expression by Western blot, and numbers of MDSCs using FACS.

## Results

### Expression of IL-13Rα2 in *Tgfbr1/Pten* 2cKO mouse with HNSCC

**Figure 1:**
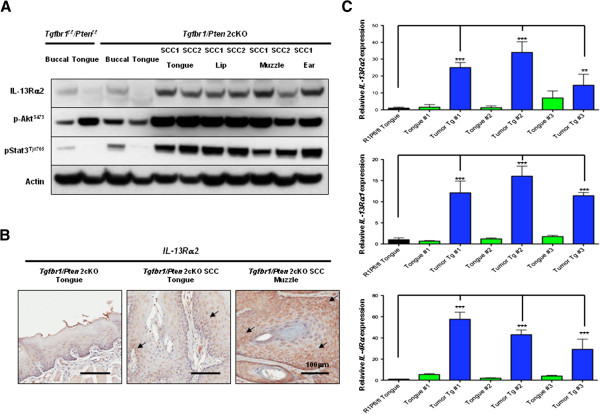
**Upregulation of IL-13Rα2 in tumors from *Tgfbr1/Pten *2cKO mice. **(**A**) Western blot analysis showing upregulation of IL-13Rα2 in multiple squamous cell carcinomas (SCC) which develop from loss of *Tgfbr1/Pten *in the head and neck epithelium. Tumors can be identified by increases in both p-Akt, genetically induced with *Pten *deletion, and in p-Stat3, a commonly activated signaling pathway in HNSCC. (**B**) Positive immunohistochemical analysis showing IL-13Rα2 staining on two representative SCCs as compared to the normal tongue epithelium. (**C**) Real-time PCR shows increases in mRNA of all IL-13 receptor subunits between three pairs of tongue tumors and the normal tongue tissue (P value < 0.0001 in most cases).

Deletion of TGFβRI and PTEN in the oral epithelium of mice has been shown to lead to the formation of spontaneous tumors that resemble human HNSCC, including similar tumor promoting alterations in various cell signaling pathways [1]. As previously reported the tumors in these mice look like human HNSCC pathologically with atypical cells and differentiated keratin pearls. The mouse tumors also mirror the same molecular alterations found in human HNSCC such as EGFR overexpression and activated Akt, NF-κB, and Stat3 signaling. Since IL-13Rα2 is over-expressed in about 33% of human HNSCCs [7], we decided to examine the tumors of these mice for this unique cancer marker. Western blot showed that IL-13Rα2 is upregulated in the tumors of the *Tgfbr1/Pten* 2cKO mice (Figure 1A). Immunohistochemistry analysis also showed staining of SCC from tongue and muzzle tumors but not in the normal tongue (Figure 1B). As previously described, HNSCC in the *Tgfbr1/Pten* 2cKO mice can be identified with activated Akt due to PTEN deletion, along with phosphorylated Stat3, both cell signaling pathways that are commonly activated in human HNSCC [1]. These two markers were used to verify that the IL-13Rα2 expression is primarily restricted to the HNSCC. The tumors that arise because of TGFβRI and PTEN deletion occur not only on the tongues of the mice, but also on the head and neck epithelium around the ears and the muzzle. Regardless of location, all squamous cell carcinomas displayed high expression of IL-13Rα2, making the mouse model ideal for studying targeted cytotoxin therapy against IL-13Rα2. Using real-time PCR, we also examined three sets of mice for the expression of transcripts for various chains of receptors, e.g. IL-13Rα2, IL-13Rα1, and IL-4Rα, which are involved in IL-13 receptor structure and function. We observed an upregulation of the transcripts of all three subunit receptors in the tongue tumors, as compared to the normal corresponding adjacent tongue tissue (Figure 1C). Expression of the IL-13 receptors was significantly higher in the tongue tumors from the *Tgfbr1/Pten* 2cKO mice (P value < 0.0001) when compared with the normal tongue tissue in which TGFBR1 and PTEN was not genetically deleted.

### Cytotoxicity of IL-13-PE on primary tumor cells from *Tgfbr1/Pten* 2cKO mice

**Figure 2:**
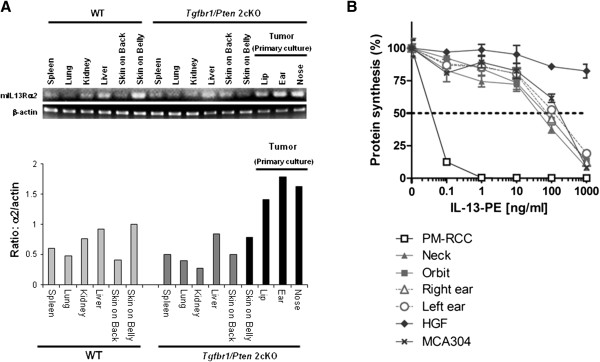
**Sensitivity of primary tumor cultures to IL-13-PE treatment. **(**A**) RT-PCR shows that IL-13Rα2 expression is high for the primary cultures but low in other tissues from *Tgfbr1/Pten *2cKO mice undergoing tumor induction. (**B**) Primary cultures are sensitive to the cytotoxic effects of IL-13-PE, as measured by a decrease in protein synthesis. An IC_50 _for IL-13-PE ranged between 45–100 ng/ml in the primary cultured tumor cells overexpressing IL-13Rα2.

Since IL-13Rα2 binds to IL-13 with high affinity and then undergoes internalization, expression of this receptor can thereby be targeted for delivery of a bacterial toxin into a cancer cell [18]. The therapeutic agent IL-13-PE was designed to bind to cells expressing IL-13 receptors and cause selective cytotoxicitiy upon internalization. Primary cultures were derived from the tumors of the *Tgfbr1/Pten* 2cKO mice to determine if the upregulated expression of IL-13Rα2 by the cancer cells would make them sensitive to the IL-13-PE cytotoxicity. The isolated primary cancer cells from the *Tgfbr1/Pten* 2cKO mice retain higher expression of IL-13Rα2 as compared to other tissues, including the spleen, lung, kidney, liver, and skin (Figure 2A). Higher expression of IL-13Rα2 is therefore primarily limited to the tumor cells and does not appear to be aberrantly triggered in any other tissues through the tumor induction process, which causes conditional deletion of TGFβRI and PTEN. As seen in Figure 2B, the cultured primary tumor cells with upregulated IL-13Rα2 were sensitive to the addition of IL-13-PE in a dose-dependent manner. Using two *Tgfbr1/Pten* 2cKO mice, each with multiple tumor sites, an IC_50_ (the concentration of drug causing 50% inhibition of protein synthesis) ranging between 45–100 ng/ml was determined for IL-13-PE. For this *in vitro* testing, PM-RCC was selected as a positive control out of many cell lines tested because of higher expression of high-affinity IL-13Rα2. Based on sensitivity (IC_50_*)* of primary cells to IL-13-PE, these cells most likely express lower number of IL-13 receptors. We have not tested affinity of IL-13-PE binding on these cells. However, in previous studies, we demonstrated that higher binding affinity of IL-13-PE to tumor cells did not enhance cytotoxicity to cells because internalization of only few molecules of IL-13-PE was enough to kill the cells [19]. The primary tumor cultures seem to have an intermediate expression of IL-13Rα2 where the IC_50_ for these cells is similar to the murine sarcoma cell line MCA304, which has moderate sensitivity to IL-13-PE [20]. Human gingival fibroblasts (HGF), which don’t display IL-13Rα2, were unaffected by IL-13-PE treatment. It is to be noted that cytotoxicity of IL-13-PE toward IL-13Rα2 positive cells is highly specific as we have shown in our previous several studies that an excess of IL-13 (100–200 fold) neutralizes activity of IL-13-PE [10,21].

### IL-13-PE treatment of *Tgfbr1/Pten* 2cKO mice

**Figure 3:**
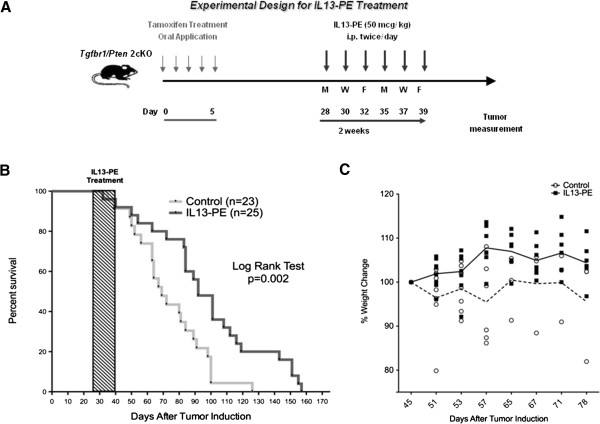
**Increased survival in *Tgfbr1/Pten *2cKO mice dosed with IL-13-PE. **(**A**) A schematic showing the dosing schedule of IL-13-PE to *Tgfbr1/Pten *2cKO mice. (**B**) Survival curve shows increased longevity in *Tgfbr1/Pten *2cKO mice treated with IL-13-PE (measured in days following tumor induction). Median survival increased from 69 days in the controls to 92 days with IL-13-PE treatment (P value = 0.002 Log-rank [Mantel-Cox] Test). (**C**) The treated mice continue gaining weight after IL-13-PE therapy as compared to the control mice (P value < 0.0001 Two-way ANOVA).

With confirmation of the cytotoxic effects of IL-13-PE on cultured primary murine HNSCC tumor cells, we next decided to treat *Tgfbr1/Pten* 2cKO mice with i.p. injections of the recombinant immunotoxin. The dosing schedule of IL-13-PE administration is illustrated in Figure 3A. Essentially, tumors were induced in mice between 5 to 7 weeks of age using daily oral administration of Tamoxifen for five days to promote the recombinase activity of the *K14-CreER*^*tam*^ transgene. The *K14-CreER*^*tam*^ in turn causes recombination in the *Tgfbr1f/f* and *Ptenf/f* alleles and disrupts the expression of these two important tumor suppressors. Treatment with IL-13-PE was then initiated four weeks from the day tumor induction began, since papillomas and early carcinomas generally start to appear at this time in the *Tgfbr1/Pten* 2cKO mouse. The *Tgfbr1/Pten* 2cKO mice were dosed with two i.p. injections of IL-13-PE (50 μg/kg) per day (b.i.d.) at a minimum interval of 6–8 hr on alternate days for two weeks. The dose selected for this study was less than the maximum tolerated dose for IL-13-PE based on several previous mouse experiments [10]. The preclinical safety studies characterizing the organ toxicities stemming from higher doses of IL-13-PE have already been described previously [22,23]. The *Tgfbr1/Pten* 2cKO mice treated with IL-13-PE (n=25) showed increased survival (p=0.002) compared to the littermate controls that were injected only with excipient (0.2% human serum albumin in PBS) (n=23) (Figure 3B). IL-13-PE was administered to the mice as they first started to succumb to tumor-associated mortality from the developing squamous cell carcinomas. The median survival significantly increased from 69 days after tumor induction in the controls to about 92 days through IL-13-PE treatment. At day 90, around the day of the median survival, 14 out of 25 (or *56%*) survived in the IL-13-PE treatment group compared to 5 out of 23 (or *21%*) in control group. More than twice as many treated mice were surviving at this time point as compared to the controls, clearly indicating the delay in progression of disease. In these survival studies, about 65% of the mice develop tongue tumors that impair the ability to eat and therefore cause cachexia. The mice given the IL-13-PE therapy had roughly the same occurrence of tongue tumors as the controls, so the immunotoxin treatment most likely delayed the development of these tumors, which accounts for the increased survival. As previously reported, metastasis was not observed in the *Tgfbr1/Pten* 2cKO cancer model because of the rapid growth and progression of the carcinomas that develop in these mice, so the effects of IL-13-PE treatment on metastasis could not be observed [1].

The IL-13-PE administration was well-tolerated by the *Tgfbr1/Pten* 2cKO mice and the mice treated with cytotoxin therapy showed improvements in their health, as opposed to the control littermates. In the few mice that had tumors at the onset of IL-13-PE treatment, the tumors appeared to regress after immunotoxin administration, in contrast to the untreated mice. Mice treated with IL-13-PE continued to gain weight while the controls lost weight due to the developing head and neck tumors (P value < 0.0001; Figure 3C). In particular, mice with carcinomas on the tongue lost weigh due to an inability to eat properly. No histological toxicity was detected in association with IL-13-PE treatment in tissues such as the spleen, lung, and heart (data not shown), which thereby demonstrated the restricted targeting of the IL-13-PE cytotoxin to only the cancer cells and not the normal tissues. The receptor-targeted specificity of IL-13-PE has been shown earlier by using an irrelevant IL2-PE immunotoxin as a control. IL2-PE, a immunotoxin known to exert its cytotoxicity through binding to IL-2 receptor γ chain was not cytotoxic to IL-13Rα2 positive tumor cells confirming that IL-13-PE-mediated cytotoxicity is IL-13Rα2 specific [24].

### Decreased expression of IL13Rα2 after IL-13-PE treatment

**Figure 4:**
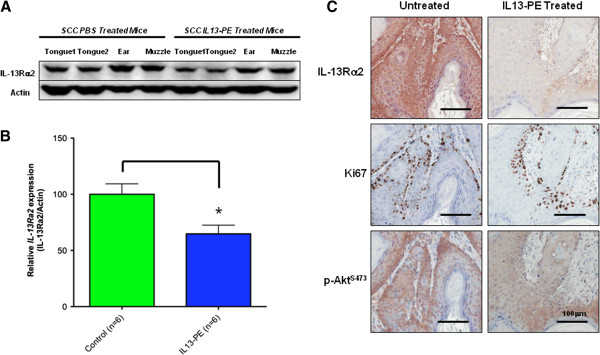
**Decreased IL-13Rα2 expression through IL-13-PE immunotoxin treatment. **(**A**) Western blot showing decreased expression of IL-13Rα2 in SCCs from IL-13-PE treated mice when compared to the corresponding control SCCs. Tumors developed from different locations in the head and neck epithelium such as the tongue, muzzle, and ear. (**B**) Tumors from the muzzle and ear of the IL-13-PE treated mice show a significant decrease (P value < 0.05) in relative IL-13Rα2 expression compared to the non-treated mice. (**C**) Immunohistochemistry shows a general decrease in IL-13Rα2 expression on a tumor from the ear of an IL-13-PE treated mouse as compared to the control but, of the cancer cells that escaped cell death by IL-13-PE, there was no change in p-Akt, a genetic change induced by *Pten* deletion, nor was there any difference in active proliferation through Ki67 staining.

Immunotoxin therapy by IL-13-PE will essentially target cancer cells that upregulate IL-13Rα2 and will therefore select against its expression. We looked at the tumor tissues from treated *Tgfbr1/Pten* 2cKO mice to see if the expression of IL-13Rα2 was decreased. Expression of IL-13Rα2 was variable in the tumors collected from the *Tgfbr1/Pten* 2cKO mice but was generally decreased overall in the IL-13-PE treated mice (Figure 4A). In the IL-13-PE treated mice, the levels of IL-13Rα2 were significantly decreased in tumors that developed on the head and neck epithelia such as the muzzle and ears (P value < 0.05; Figure 4B), but no significant difference was detected for squamous cell carcinomas on the tongue (data not shown). Decreased expression of IL-13Rα2 in the tumors was also detected through immunohistochemical analysis (Figure 4C) but, of the cancer cells that evaded cell death by IL-13-PE, no difference in proliferation could be seen with Ki67 staining as compared to the untreated mice. In addition, IL-13-PE had no significant effect on phosphorylation of Akt, a tumor promoting kinase that is irreversibly activated in the *Tgfbr1/Pten* 2cKO mice due to genetically Cre-mediated PTEN deletion. No association could be made between the level of IL-13Rα2 on a tumor and the time since administering IL-13-PE treatment. Treatment with IL-13-PE at an early time point in cancer development may have selectively hindered the growth and establishment of high IL-13Rα2 expressing cancer cells in the mice, resulting in the formation of tumors with decreased overall expression of this receptor.

### Reduction in MDSCs but not Tregs through IL-13-PE treatment

**Figure 5:**
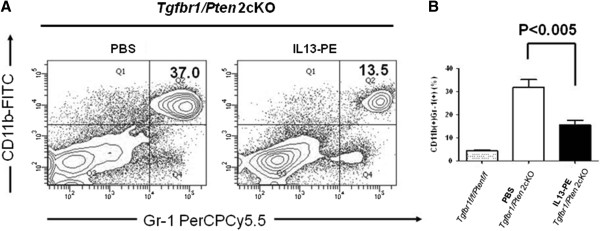
**MDSCs were decreased by IL-13-PE treatment in *Tgfbr1/Pten *2cKO mice. **(**A**) Representative flow profile showing decreased populations of CD11b+Gr-1+ cells through IL-13-PE treatment. (**B**) Quantification of flow cytometry results. IL-13-PE treated mice are compared with both the saline injected *Tgfbr1/Pten *2cKO tumor-bearing controls and the *Tgfbr1f/f/Ptenf/f* mice without tumors. MDSCs were significantly reduced with IL-13-PE treatment (P value < 0.005).

The expression of IL-13Rα2 has been linked to deleterious immune effect such as tumor immune evasion, particularly since signaling through this receptor causes upregulation of the immunosuppressive cytokine TGF-β1 [11,12]. To examine for any immunological changes in the treated mice, some animals were sacrificed three days after the last dose of IL-13-PE, and FACS analysis was performed using the splenocytes. The MDSCs, a heterogeneous population of myeloid cells known to promote tumor immune evasion, were detected with FACS analysis using the monocyte/macrophage markers CD11b+ and the granulocyte antigen Gr-1+ [17]. The *Tgfbr1f/f/Ptenf/f* mice lacking the *K14-CreER*^*tam*^ transgene to cause conditional deletion were also analyzed as normal controls without tumors. As expected, the numbers of MDSCs in the spleens increased from about 4.3 % in the *Tgfbr1f/f/Ptenf/f* mice to 32% in the untreated *Tgfbr1/Pten* 2cKO mice due to deletion of TGFβRI and PTEN and to the subsequent tumor development. Interestingly, the number of MDSCs dropped by half to 15.5% (P < 0.005) with IL-13-PE treatment (Figure 5B). A representative FACS plot is shown in Figure 5A*.*

Although the number of MDSCs decreased with IL-13-PE treatment, no significant change was seen in the number of macrophages from the spleens of the mice (data not shown). Another immunosuppressive population of cells, T regulatory cells (Tregs), also showed no change between the two groups of mice (data not shown). The MDSCs, however, may be particularly sensitive to IL-13-PE treatment since IL-13Rα2 has been shown to express on some populations of these myeloid cells in order to induce TGF-β1 secretion and promote tumor immune evasion [11].

## Discussion

In order to aid in the discovery of new treatments and mechanisms of human HNSCC actions, we have developed a mouse model that spontaneously forms tumors in the head and neck epithelium with 100% penetrance. We observed that these tumors mimic human HNSCC with similar morphology and altered cell signaling. With such resemblance to human HNSCCs, the *Tgfbr1/Pten* 2cKO mice could be useful for screening novel therapeutics against cancer. Cancer cells often overexpress cell surface receptors of both growth factors and cytokines that help promote tumor growth, proliferation, and immune evasion. As previously shown, IL-13Rα2 is upregulated in about 33% of human HNSCC [7] but remains absent or shows very low expression on normal cells. Our previous *in vitro* studies showed that IL-13Rα2-positive HNSCC cell lines were sensitive to cytotoxic effect of IL-13-PE depending upon the level of their expression of IL-13Rα2 chain. The specificity and requirement of IL-13Rα2 chain for cytotoxicity was further confirmed by transient transfection of IL-13Rα2 chain in two IL-13Rα2-negative HNSCC cell lines. Both transfectants acquired sensitivity to IL-13-PE confirming the relevance of the utility of IL-13-PE in head and neck cancer therapy [25]. We demonstrate that tumors from *Tgfbr1/Pten* 2cKO mice express high levels of not only IL-13Rα2, but of IL-13Rα1 and IL-4Rα as well. We have previously shown that cultured cells derived from the periorbital and perianal squamous cell carcinomas collected from another TGFβRI conditional knockout model can also express IL-13Rα2 and are sensitive to IL-13-PE treatment [16]. Future studies will be conducted to determine if TGFβRI deletion facilitates IL-13Rα2 expression on squamous cell carcinomas. Because cell surface receptors can be targeted with monoclonal antibodies and fusion toxins, we tested whether the expression of the IL-13 receptors in tumors derived from *Tgfbr1/Pten* 2cKO mice can be targeted as part of a strategy to treat HNSCC. We found that a recombinant IL-13-PE immunotoxin targeting IL-13Rα2 has remarkable anti-cancer activity against these tumors.

IL-13-PE immunotoxin is specifically designed to target tumors expressing this receptor by combining the IL-13 cytokine with the *Pseudomonas* exotoxin [9]. Bacterial toxins are ideal for generating immunotoxins since they can be easily produced in *E. coli* and show few side effects in humans if properly targeted [26]. Upon endocytosis, the fusion toxin translocates to the cytosol and inactivates elongation factor 2 to cause cell death. IL-13Rα2 is also an ideal target for cytotoxin-based therapy since it is a unique tumor target displaying a high affinity for IL-13 and undergoes endocytosis upon binding. These qualities minimize the uptake of IL-13-PE by normal cells. Targeting IL-13Rα2 expression may also be useful in restoring proper immunosurveillance [11].

As an anti-cancer strategy, IL-13-PE immunotoxin treatment has already proven to be efficacious against tumors in many cancer mouse models and is currently being used in clinical trials. IL-13-PE was used in a Phase 3 clinical study to treat patients with glioblastoma multiforme, another cancer type wherein IL-13Rα2 is overexpressed in the majority of tumor cells. Convection-enhanced delivery of IL-13-PE was well-tolerated by patients with malignant gliomas [27]. One challenge of immunotoxin and any targeted therapy is efficient penetration into solid tumors.

Nevertheless, in the *Tgfbr1/Pten* 2cKO mouse model of HNSCC, systemic administration of IL-13-PE resulted in significantly increased survival in the treated mice as compared to the untreated controls. Tumor regression was evident in the few mice that had visible tumors at the start of IL-13-PE, particularly in the SCCs around the muzzle. Unlike the controls, the mice treated with the IL-13-PE group did not experience the immediate weight loss that typically occurs during tongue tumor development due to obstructed eating. However, both groups of mice in the survival studies eventually had the same occurrence of SCCs on the tongue, suggesting a possible delay in the formation and growth of these tumors. The weight gain together with the lack of any histological signs of toxicity also demonstrate that the IL-13-PE treatment is well-tolerated by the mice. In our recent study on the role of IL-13Rα2 in pancreatic cancer invasion and metastasis, we demonstrated that IL-13Rα2–positive cancer metastasized to lymph nodes, liver, and peritoneum at a significantly higher rate compared with IL-13Rα2–negative tumors. The level of IL-13Rα2 expression in metastatic lesion was higher compared to the primary tumors [28]. Though we did not investigate the presence of metastasis in this model, the pancreatic tumor cells collected from lymph nodes metastasis were much more sensitive to IL-13-PE in vitro than compared with primary tumor cells indicating higher level of IL-13Rα2 in metastatic lesion. Based on this data, we can predict similar efficacy of IL-13-PE to eliminate metastatic lesions common in patients with HNSCC. Future studies will be conducted to determine the optimal schedule and dose of administration of IL-13-PE.

It is interesting to note that IL-13Rα2 expression was decreased in the tumors of IL-13-PE treated mice, indicating that high IL-13Rα2 positive cells are eliminated while IL-13Rα2 negative or low expressing tumors were not. However, this selective depletion of tumor cells did not cause any impact on proliferation of tumor cells as no difference in Ki67 staining was observed in tumors of treated mice compared to those of the untreated mice.

To examine the mechanism of antitumor response by IL-13-PE, we investigated whether treatment affected immunosurveillance to subsequently allow for enhanced tumor destruction by the immune system. IL-13-PE treatment caused a significant decrease in MDSCs in the spleen of treated mice that corresponded with increased survival. On the other hand, another immunosuppressive population of cells, Tregs, showed no change in the spleens between the two groups of mice (data not shown). This may be due to a direct cytotoxic effect of IL-13-PE on MDSCs, since some of these myeloid cells are known to express IL-13Rα2 in order to induce TGF-β1 secretion and promote tumor immune evasion [12]. Future studies will examine the status of MDSCs and T regs in various tumors of these mice. However, it is possible that the reduction in the number of MDSCs resulted in improved immunosurveillance and, consequently, enhanced survival of the animals.

Taken together, the results from the *Tgfbr1/Pten* 2cKO mice validate our previous report on nude mice with transplanted tumors, which suggests that IL-13-PE may be an effective therapeutic agent for the treatment of HNSCC. Along with delaying tumor formation, we were able to see in the immunocompetent *Tgfbr1/Pten* 2cKO mice that the IL-13-PE treatment could additionally limit the development of an immunosuppressive tumor environment. Other treatment delivery options for IL-13-PE could improve the effectiveness of this immunotoxin in the *Tgfbr1/Pten* 2cKO mice such as direct injection into the head and neck tumors or a surgically implanted continuous infusion pump [22]. IL-13-PE has also been shown to work synergistically with paclitaxel in an immunodeficient animal model of HNSCC [29] with gemcitabine for pancreatic cancer [30] and a DNA vaccine of IL-13Rα2 in melanoma, breast, and sarcoma tumor models [20]. It is therefore possible that any of these combinations would be useful for testing the *Tgfbr1/Pten* 2cKO mouse model of spontaneous cancer. Further studies will be performed to test this combination approach. In summary, our study demonstrates that our HNSCC mouse model is valuable for developing novel cancer therapeutic approaches [31], and that IL-13-PE has therapeutic potential to treat human head and neck cancer.

## Conclusion

Conditional deletion of both TGFβRI and PTEN in the oral cavity of mice results in the formation of spontaneous squamous cell carcinomas in the head and neck area that display IL-13Rα2, a tumor antigen expressed in 33% of human HNSCCs. Primary cultures from the *Tgfbr1/Pten* 2cKO mouse tumors show sensitivity to IL-13-PE. The *Tgfbr1/Pten* 2cKO mice were therefore tested with IL-13-PE (50 μg/kg b.i.d. on alternate days) for two weeks starting at an early point in tumor induction when carcinomas are first beginning to appear. This IL-13-PE regimen significantly increased survival in the tumor bearing mice (P value 0.002) with no signs of toxicity. Expression of IL-13Rα2 was reduced in the tumors while, concurrently, the spleens of the treated mice show reduced numbers of MDSCs, a population of myeloid cells that aid in tumor immune evasion. The increased survival of the IL-13-PE treated mice helps to further validate the idea that targeted therapy against IL-13Rα2 expression could be employed as a clinical means of inhibiting HNSCC.

## Competing interests

The authors declare that they have no competing interests.

## Authors’ contributions

Conception and design: BH, HN, Z-JS, SRH, RKP, ABK. Development of methodology: BH, HN, SRH, YB, Acquisition of data (provided animals, acquired and managed patients, provided facilities, etc.): BH, HN, Z-JS, YS, ABK, Analysis and interpretation of data (e.g., statistical analysis, biostatistics, computational analysis): BH, HN, SRH, RKP, ABK, Writing, review, and/or revision of the manuscript: B. Hall, S.R. Husain, R.K. Puri, A.B. Kulkarni, Administrative, technical, or material support (i.e., reporting or organizing data, constructing databases): SRH, YB, Study supervision: RKP, ABK. All authors read and approved the final manuscript.
